# Technological and socioeconomic characteristics of smallholder dairy farms in Indigenous Pastos communities of Colombia

**DOI:** 10.1007/s11250-025-04576-4

**Published:** 2025-08-11

**Authors:** Juan P. Taramuel-Taramuel, Miguel Augusto Delgado-López, Omar E. Aza-Fuelantala, Dursun Barrios

**Affiliations:** 1 Facultad de Ciencias Agrarias, Grupo de Investigación Biogénesis, Universidad Nacional de Colombia, Bogotá D.C., Colombia; 2 Facultad de Ciencias de la Administración, Universidad del Valle, Cali, Colombia

**Keywords:** Dairy cooperatives, Dairy farming, Indigenous people, Smallholder agriculture

## Abstract

Indigenous dairy farming systems in Latin America represent an important but understudied sector where traditional agricultural practices intersect with modern technology adoption. This study examined technological adoption patterns and socioeconomic characteristics of smallholder dairy farms in indigenous Pastos communities of Cumbal, Nariño, Colombia. Data was collected from 542 dairy farms across three communities (Cumbal, Panan, and Chiles) using structured questionnaires. Multiple correspondence analysis (MCA) and hierarchical cluster analysis (HCA) revealed two distinct groups: Technology-intensive adopters (68.8%) and Technology-traditional adopters (31.2%). The first group showed significantly higher rates of cooperative membership (88.2% vs. 40.2%, *p* < 0.001), training participation (82% vs. 43.2%, *p* < 0.001), and implementation of practices such as the use of protein, energy, and mineral supplements (98.4% vs. 81.7%, *p* < 0.01), pasture fertilization (89.3% vs. 66.9%, *p* < 0.01), and artificial insemination (76.7% vs. 40.8%, *p* < 0.001). Geographic location significantly influenced adoption patterns (*p* < 0.001), with farms closer to urban centers showing higher technology adoption rates. Technology-intensive adopters operated significantly larger farms (3.36 vs. 2.8 hectares on average, *p* < 0.01) compared to Technology-traditional adopters and achieved significantly higher milk production (7.94 vs. 7.25 L per cow per day, *p* < 0.01) and derived greater income from dairy activities (*p* < 0.001). The findings suggest the need for differentiated support programs that consider both spatial and cultural dimensions when promoting dairy sector development in indigenous territories. Future research should focus on cost-benefit analyses of adopted technologies, cultural factors influencing adoption decisions, and environmental impacts of dairy intensification in ecologically sensitive indigenous territories.

## Introduction

Dairy farming is a crucial economic activity in Colombia, contributing 1.7% to the national Gross Domestic Product (GDP), 20.2% to the agricultural GDP, and 51.5% to the livestock GDP (Federeación Colombiana de Ganaderos (FEDEGAN), 2024). The country has over 620,000 livestock farms, with about 80% being small farms holding less than 50 animals (Durana et al. 2023). As the fourth-largest milk producer in Latin America, producing 7,251 million liters in 2022, the dairy sector provides around 700,000 direct jobs (Ministry of Agriculture and Rural Development 2023).

In the department of Nariño, dairy farming is particularly significant, providing around 160,000 direct jobs, accounting for 27% of the agricultural GDP, and comprising approximately 31,000 smallholder dairy farms (Agencia de Desarrollo Rural (ADR) & Food and Agriculture Organization (FAO), 2019; Government of Nariño 2020; Instituto Colombiano Agropecuario (ICA), 2023). The municipality of Cumbal, located in the Andean region of Nariño, is characterized by small-scale dairy operations averaging 6 animals per farm (ICA, 2023). The area includes the páramo Cumbal-Chiles complex, where livestock expansion has raised environmental concerns about this fragile Andean ecosystem (Zapata et al. 2021; Durana et al. 2023).

Cumbal is an Indigenous territory encompassing four communities (Cumbal, Panan, Chiles, and Mayasquer) of the Pastos people (Rappaport 1988). It is Colombia’s fourth-largest indigenous group with approximately 164,000 members (Departamento Administrativo Nacional de Estadística (DANE), 2019). Like many indigenous communities that depend on agriculture for their livelihoods (Pérez-Silva et al. 2023), the Pastos in Cumbal derive their primary income from dairy farming (Government of Nariño 2020).

The region has been the target of public and private interventions aimed at improving animal feed practices, promoting dairy farmer associations, building milk collection centers, expanding market linkages, disseminating artificial insemination, and enhancing farm management practices (United States Agency for International Development (USAID), 2010; Thomson and Jaramillo 2017; ADR & FAO, 2019). However, significant challenges persist, including low productivity, limited technological adoption, insufficient technical assistance, and restricted credit access (Government of Nariño 2024).

Understanding these adoption challenges requires recognizing that agricultural technology adoption is embedded within complex socioeconomic, cultural, and geographical contexts. The diffusion of innovations theory (Rogers 2003) provides a valuable framework for understanding how new technologies spread through social systems and it identifies key adopter categories based on innovation acceptance timing. This framework helps explain the heterogeneous technology adoption patterns commonly observed in agricultural communities, particularly in indigenous contexts where traditional practices remain strong. Additionally, the technology acceptance model (Davis 1989) proposes that perceived usefulness and ease of use significantly influence farmers’ decisions to adopt new technologies, particularly in smallholder contexts where risk aversion is prevalent due to limited resources and high vulnerability to production failures.

Farmers commonly adopt technologies and management practices to enhance farm outcomes, including increased output yield, profitability, and productivity (Läpple and Thorne 2019; Nnahiwe et al. 2023). Studies have shown that technological innovations positively influence productive and economic performance, making them key drivers of farm viability (De-Pablos-Heredero et al. 2020). Farmers exhibiting greater adoption levels tend to achieve higher milk production (Gebre et al. 2022).

Farm typological classification based on technological level, production structure, and management practices provides a starting point for implementing strategies to improve economic viability. Understanding the factors influencing technology adoption is crucial for designing effective private and public interventions (Riveiro et al. 2013).

While extensive research exists on agricultural technology adoption, there is limited documentation of how indigenous communities in Latin America engage with and implement new agricultural technologies, including their unique motivations, constraints, and cultural considerations (Sellers and Bilsborrow 2019). The Pastos communities represent a particularly interesting case, as they have maintained traditional agricultural practices while gradually incorporating modern dairy farming techniques/practices.

The objectives of this study were to: (i) identify and characterize homogeneous groups of dairy production systems in indigenous Pastos communities based on technological adoption patterns; (ii) analyze the socioeconomic factors that influence technology adoption among indigenous dairy farmers; and (iii) explore associations between technology adoption patterns and production levels. This research aims to provide useful information to guide public and private interventions, particularly as the Government of Nariño has announced plans to strengthen the implementation of modern technologies in the agricultural sector to improve productivity and efficiency in production (Government of Nariño 2024).

## Materials and methods

### Study area

**Fig. 1:**
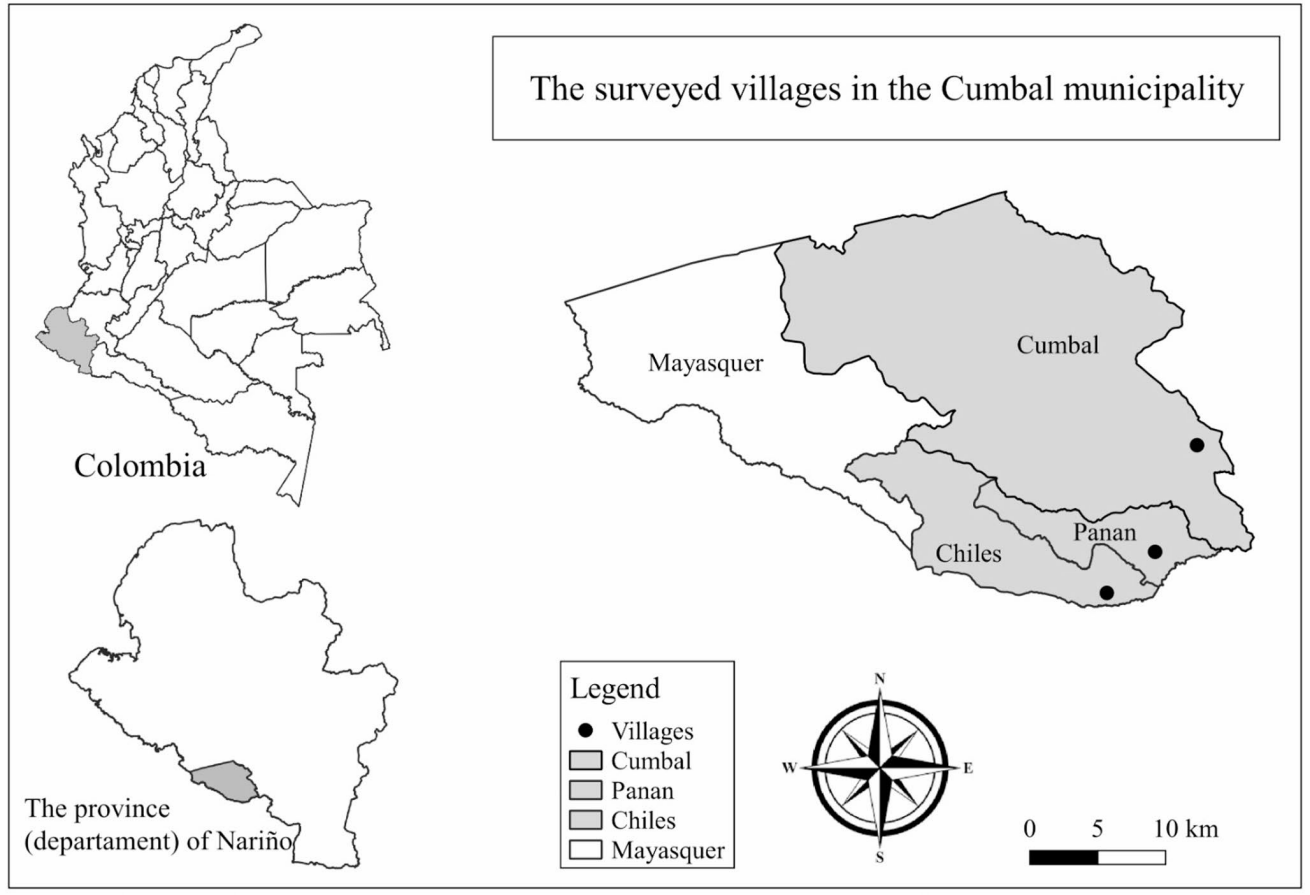
Location map of field sampling communities in the department (province) of Nariño, Colombia

The study was conducted in three Pastos communities: Cumbal, Panan, and Chiles, within the municipality of Cumbal, department of Nariño, southwestern Colombia (Fig. 1). Dairy production systems in the municipality are based mainly on grazing located in the Andean highlands and slopes over 3,000 m above sea level. Milk is sold to local small dairy processing companies and intermediate sellers or larger dairy processing enterprises such as Alpina, Alquería, or Alival (Aza 2021).

### Field sampling

Data was collected in 2023 from 542 dairy farms (Cumbal = 444, Panan = 48, and Chiles = 50) with a total of 4,078 milking cows (233 cows from Chiles, 3,537 cows from Cumbal, and 308 cows from Panan), averaging approximately 8 cows in production per farm. The dairy herds consisted mainly of *Bos taurus* dairy breeds, including Holstein, Jersey, Brown Swiss, and their crossbreeds, as well as Criollo cattle. A non-probability sampling was conducted using a purposive sampling approach to capture the diversity of dairy farms in the study region (Saunders et al. 2023). Three specific inclusion criteria were employed: (1) only active dairy farms with milking cows at the time of the survey were included; (2) respondents had to be primary decision-makers for the farm operation; and (3) farms spanning different sizes were deliberately included to represent various production scales and management practices. The sample size represents approximately 13% of the dairy farms in the study area (ICA, 2023).

While purposive sampling allowed us to target the specific population of interest, we acknowledge certain limitations associated with this approach. As a non-probability sampling method, it introduces potential selection bias and limits the generalizability of findings to the broader population (Etikan et al. 2016). To mitigate these limitations, we ensured geographical representation across all three communities and included farms of various sizes and production systems.

### Sampling tools

A structured questionnaire was designed to gather information on socioeconomic characteristics, farm characteristics, technologies, and farm management practices. To establish farm typology, 22 qualitative variables were selected from the survey addressing important characteristics related to technology adoption (Table 1). The data collected was anonymous, and the survey was voluntary.

**Table 1:** Variables, codes, and modalities used for multiple correspondence analysis of cumbal’s dairy farming practices and socioeconomic characteristics

Variables	Codes	Modalities
Village	VILL	Chiles
		Cumbal
		Panan
Age	AGE	Less_40yrs
		Between_40-60yrs
		More_60yrs
Gender	GEN	M = Male
		F = Female
Experience in dairy farming (in years)	EXP	ExpLess_20
		Exp_Between_20–40
		ExpMore_40
Education level	EDU	EDU_1 = Incomplete elementary school
		EDU_2 = Elementary school
		EDU_3 = High school
		EDU_4 = Technical school
		EDU_5 = University
Dairy cooperative member	COM	COM_YES
		COM_NOT
Dairy livestock training	TRA	TRA_YES
		TRA_NOT
Area in hectares (1 hectare = 2.471 acres)	HEC	HecLess_3
		Hec_Between_3–6
		HecMore_6
Daily milk production per cow	PROD	ProdLess_5L
		Prod_Between_5L-15 L
		ProdMore_15L
Household income from dairy activity	INC	INC_1 = Less or equal than one quarter (25%)
		INC_2 = Less or equal than half (50%)
		INC_3 = Less or equal than three quarters (75%)
		INC_4 = Total (100%)
Main economic activity	MAI	MAI_1 = Other non-agricultural activities
		MAI_2 = Other agricultural activities
		MAI_3 = Dairy production
Portable milking machine	MLK	MLK_YES
		MLK_NOT
Artificial insemination	IAR	IAR_YES
		IAR_NOT
Electric fence to pasture (portable)	FEN	FEN_YES
		FEN_NOT
Use improved pastures (Raygrass, Clover)	PAS	PAS_YES
		PAS_NOT
Pasture fertilization	FER	FER_YES
		FER_NOT
Protein, energy and mineral supplements for cows	SUE	SUE_YES
		SUE_NOT
Health and reproductive records	REH	REH_YES
		REH_NOT
Implementation of Good Farming Practices	GFP	GFP_YES
		GFP_NOT
Bookkeeping software (specialized software, spreadsheet)	BFT	BFT_YES
		BFT_NOT
Production cost analysis	COA	COA_YES
		COA_NOT
Smartphone to search farming information	SMT	SMT_YES
		SMT_NOT

Note: VILL = Village; AGE = Age; GEN = Gender; EXP = Experience in dairy farming; EDU = Education level; COM = Dairy cooperative member; TRA = Dairy livestock training; HEC = Area in hectares; PROD = Daily milk production per cow; INC = Household income from dairy activity; MAI = Main economic activity; MLK = Portable milking machine; IAR = Artificial insemination; FEN = Electric fence to pasture; PAS = Use improved pastures; FER = Pasture fertilization; SUE = Protein, energy and mineral supplements for cows; REH = Health and reproductive records; MCA = Multiple Correspondence Analysis

To ensure reliability and validity of the questionnaire, we conducted pilot testing with 10 dairy farmers to refine questions for clarity and relevance. Since our questionnaire utilized binary (Yes/No) responses rather than scaled measures, traditional metrics like Cronbach’s alpha were not applicable. Instead, we employed field cross-verification techniques.

### Statistical analysis

Data processing and analysis were performed using R software with the FactoMiner package (R Core Team 2023). Following the methodological approach for dairy systems characterization outlined by Paredes et al. (2024), multiple correspondence analysis (MCA) followed by hierarchical cluster analysis (HCA) were performed to identify homogeneous groups. The data obtained from the clusters were represented using dendrograms to identify different levels of grouping based on similarity between observations (Ventocilla and Riveiro 2020).

For statistical testing, Chi-square tests were used to evaluate dependence between clusters and qualitative variables when all expected frequencies were greater than 5. When this assumption was not met, Fisher’s exact test was employed as an alternative (these are two distinct tests used under different conditions). The Kruskal-Wallis test, a non-parametric alternative to one-way ANOVA, was employed to compare quantitative variables between clusters. For all analyses, statistical significance was determined at *p* < 0.05.

Data quality control procedures included: (1) double-entry verification for 10% of questionnaires to check for transcription errors; (2) outlier detection and verification; (3) missing data analysis, with cases missing more than 20% of responses excluded from analysis (22 observations were removed from the initial database); and (4) normality testing for quantitative variables to determine appropriate statistical tests.

## Results

### Descriptive analysis

Milk production systems in the indigenous communities of Cumbal, Chiles, and Panan exhibited characteristics typical of smallholder dairy operations. The production scale was modest, with 83% of farms yielding between 3 and 300 L of milk per day. Farm sizes were generally small, with 87% of farms being 6 hectares or less. The average milk production per farm was around 58 L.

Dairy production was reported as the primary economic activity by 71% of farmers. Farmers were predominantly middle-aged, with 51% between 40 and 60 years old, and possessed substantial experience, as 52% had been engaged in dairy farming for 20–40 years. High rates of cooperative membership (73%) and participation in dairy livestock training (75%) were observed.

The adoption of basic practices was widespread, with portable electric fencing (93%), improved pastures (94%), and animal nutrition supplements (93%) being commonly implemented. However, the adoption of advanced technologies remained low, with only 10% utilizing portable milking machines or bookkeeping software. Pasture fertilization and artificial insemination showed moderate adoption rates at 82% and 65% respectively.

### Multiple correspondence analysis

**Fig. 2:**
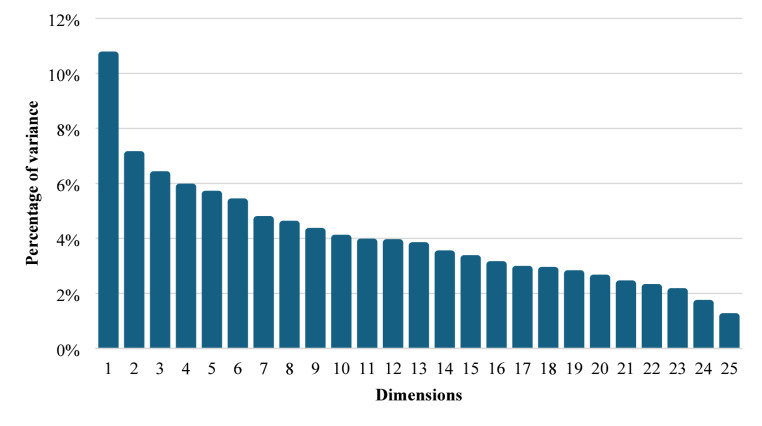
Multiple correspondence analysis of surveyed dairy farmers in Cumbal municipality, Colombia

From the MCA (Fig. 2), only the first two dimensions were retained (17.72% of the total variance) because each additional dimension contributed little to the total variance. The first dimension (10.67%) represented farms that had not received training in agricultural production, did not belong to cooperatives, and did not implement practices such as artificial insemination, fertilization of pastures, or electric fences. The second dimension (7.05%) grouped farms located in the municipality of Chiles, where smartphone use for agricultural information was limited, and whose main activity focused on non-agricultural activities.

The analysis revealed distinct patterns in technology adoption associated with geographic location and organizational membership. Distance from urban centers strongly correlated with technology adoption levels (*p* < 0.001). Farms in Cumbal and Panan communities demonstrated consistently higher adoption rates across multiple technologies compared to the more remote Chiles community.

Organizational membership, particularly participation in cooperatives, emerged as another significant factor (*p* < 0.001). Cooperative membership strongly associated with higher adoption rates of artificial insemination, pasture management practices, and animal nutrition technologies. The interaction between geographic location and organizational membership was particularly noteworthy, with cooperative membership appearing to partially mitigate geographic isolation effects.

### Hierarchical cluster analysis

**Fig. 3:**
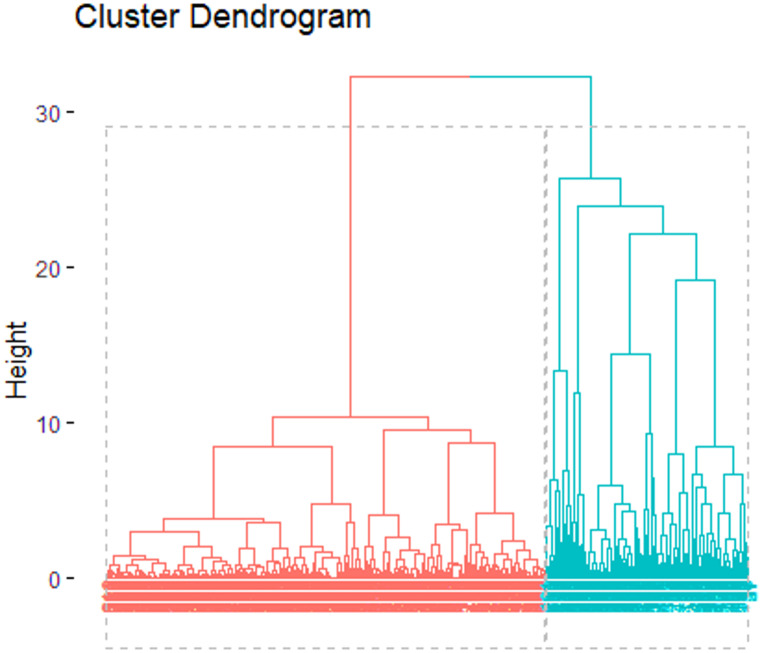
Clusters by technological adoption groups of dairy farms in Cumbal, Nariño, Colombia

The HCA revealed two distinct clusters (Fig. 3) within the indigenous communities: “Technology-intensive adopters” comprising 68.8% (*n* = 373) of surveyed farmers with 3,054 milking cows, and “Technology-traditional adopters” representing 31.2% (*n* = 169) with 1,024 milking cows. Statistical analysis showed significant differences between clusters for most variables studied (Tables 2 and 3).

**Table 2:** Distribution of cumbal’s dairy farmers by socioeconomic characteristics across two clusters identified through hierarchical classification analysis

Variables and modalities	Cluster 1 (*n* = 373)		Cluster 2 (*n* = 169)		Total	*p*-value
	*n*	%	*n*	%	*n*	
**Village**						***
Chiles	5	1.34%	45	26.63%	50	
Cumbal	321	86.06%	123	72.78%	444	
Panan	47	12.60%	1	0.59%	48	
**Dairy cooperative member**						***
COM_YES	329	88.20%	68	40.24%	397	
COM_NOT	44	11.80%	101	59.76%	145	
**Dairy livestock training**						***
TRA_YES	336	90.08%	73	43.20%	409	
TRA_NOT	37	9.92%	96	56.80%	133	
**Household income from dairy activity**						***
INC_1	7	1.88%	43	25.44%	50	
INC_2	160	42.90%	24	14.20%	184	
INC_3	97	26.01%	40	23.67%	137	
INC_4	109	29.22%	62	36.69%	171	
**Artificial insemination**						***
IAR_YES	286	76.68%	69	40.83%	355	
IAR_NOT	87	23.32%	100	59.17%	187	
**Electric fence to pasture (portable)**						**
FEN_YES	366	98.12%	141	83.43%	507	
FEN_NOT	7	1.88%	28	16.57%	35	
**Pasture fertilization**						**
FER_YES	333	89.28%	113	66.86%	446	
FER_NOT	40	10.72%	56	33.14%	96	
**Protein**,** energy**,** and mineral supplements for cows**						**
SUE_YES	367	98.39%	138	81.66%	505	
SUE_NOT	6	1.61%	31	18.34%	37	

Note: COM = Dairy cooperative membership; TRA = Dairy livestock training; INC = Household income from dairy activity (INC_1 = Less or equal than one quarter (25%), INC_2 = Less or equal than half (50%), INC_3 = Less or equal than three quarters (75%), INC_4 = Total (100%); IAR = Artificial insemination; FEN = Electric fence in pasture; FER = Pasture fertilization; SUE = Protein, energy, and mineral supplements for cows. Chi-square test was used to compare distributions between clusters. NS = No significant

** *p* < 0.01; *** *p* < 0.001

**Table 3:** Descriptive statistics of quantitative variables for cumbal’s farmers and farms characteristics across two clusters identified by hierarchical classification analysis

Items	Cluster 1 (*n* = 373)						Cluster 2 (*n* = 169)					*p*-value
	Mean	Median	SD	Min	Max		Mean	Median	SD	Min	Max	
Age (years)	46.90	47.00	13.12	20.00	80.00		48.07	47.00	13.70	20.00	79.00	NS
Experience (years)	25.46	20.00	13.80	1.00	68.00		23.06	20.00	14.55	1.00	65.00	*
Farm size (Ha)	3.36	3.00	2.30	0.50	15.00		2.80	2.00	3.17	0.50	35.00	**
Cow production (L/cow/day)	7.94	7.50	3.42	1.33	33.33		7.25	7.00	2.87	0.60	15.25	**

Note: SD = Standard deviation; Min = Minimum value; Max = Maximum value; Ha = Hectares; L/cow/day = Liters per cow per day. Age is measured in years; Experience in dairy farming is measured in years; Area is measured in hectares; Daily milk production is measured in liters per cow per day. Statistical comparisons between clusters were performed using Mann-Whitney U test for non-parametric data. NS = Not significant

* *p* < 0.05; ** *p* < 0.01; *** *p* < 0.001

### Cluster 1: Technology-intensive adopters (*n* = 373, 68.8%)

Most farmers from Cumbal (72%) and Panan (89%) communities belonged to this cluster, while only 10% from Chiles community were included (*p* < 0.001). These farmers demonstrated strong commitment to collective action, with 88.2% being cooperative members (*p* < 0.001) and 82% participating in dairy livestock training programs (*p* < 0.001). The cluster showed high implementation rates of artificial insemination (76.7%, *p* < 0.001), pasture fertilization (89.3%, *p* < 0.01), electric fence pastures (72%, *p* < 0.01), and protein, energy, and mineral supplements (98.4%, *p* < 0.01).

Farmers in this cluster averaged 46 years of age with 25.46 years of experience (*p* < 0.05). They operated larger farms (average 3.36 hectares, *p* < 0.01) and achieved higher production levels (averaging 7.94 L of milk per day, *p* < 0.01). Income dependence on dairy activity was higher, with most farmers reporting dairy income above 25% of total household income (*p* < 0.001).

### Cluster 2: Technology-traditional adopters (*n* = 169, 31.2%)

This cluster represented more traditional approaches to dairy farming, with 90% of Chiles community farmers belonging to this group (*p* < 0.001). These farmers showed low rates of cooperative membership (40.2%, *p* < 0.001) and limited training participation (43.2%, *p* < 0.001). Technology adoption was notably reduced, with artificial insemination at 40.8% (*p* < 0.001), electric fence usage at 20% (*p* < 0.01), pasture fertilization at 66.9% (*p* < 0.01), and nutritional supplements at 81.7% (*p* < 0.01).

Farmers averaged 48.07 years of age with 23.06 years of experience (*p* < 0.05). Farms were generally smaller (average 2.80 hectares, *p* < 0.01) with lower milk production (average 7.25 L per day, *p* < 0.01) compared to the high adopter group. Most farmers whose total income depends less than 25% on dairy activity were in this cluster (*p* < 0.001). The cluster showed greater variability in farm sizes, with a higher maximum (35 hectares) but lower mean and median compared to Cluster 1.

The analysis of production levels between clusters revealed significant differences in efficiency and output (*p* < 0.01). Technology-intensive adopters consistently achieved better production outcomes compared to Technology-traditional adopters. The geographic distribution of clusters highlighted significant associations between proximity to urban centers and technology adoption patterns (*p* < 0.001).

## Discussion

While extensive research has examined dairy farming technology across diverse contexts—including industrialized and developing economies, varying production scales, and different geographic regions and altitudes (e.g., Läpple and Thorne 2019; De-Pablos-Heredero et al. 2020; Gebre et al. 2022; Korir et al. 2023), studies investigating indigenous dairy farming practices and technology adoption remain scarce. This research gap is particularly notable in Latin America (Sellers and Bilsborrow 2019), where many indigenous communities rely heavily on agriculture for their economic survival (Pérez-Silva et al. 2023). This study addresses this critical knowledge gap by examining indigenous dairy farming practices and technology adoption in Cumbal, Nariño, Colombia.

Indigenous dairy farms in this territory operate as small-scale enterprises, consistent with smallholder dairy operations described by Ramírez-Rivera et al. (2019) and Silveira et al. (2022). Technology adoption in Cumbal’s dairy farms is shaped by multiple interrelated factors, including farmers’ socioeconomic characteristics and farm structural elements, consistent with García et al. (2012) findings. The dimensionality reduction approach in MCA follows best practices in agricultural systems research (Husson et al. 2017), while the clustering methodology aligns with frameworks used in previous dairy system typologies research (Paredes et al. 2024).

Geographic location significantly influenced technology adoption, with farmers in Cumbal and Panan communities showing higher adoption rates compared to those in Chiles community. Distance from urban areas restricts both market access and information access, consistently correlating with lower technology adoption rates (Mwanga et al. 2019; Akzar et al. 2022). This spatial dimension suggests that proximity to markets, infrastructure, and information sources plays a critical role in farmers’ ability to implement new technologies (Martínez-García et al. 2015).

Research on age as a determinant of agricultural technology adoption presents mixed findings. While studies by Gillespie et al. (2014) and Akzar et al. (2022) found younger farmers more likely to adopt new technologies, our results align with Gebre et al. (2022) and Korir et al. (2023), showing minimal age effects in indigenous communities. This suggests that structural factors— including geographic location, cooperative membership, and access to training— may be more influential determinants of adoption than age alone.

Farming experience significantly influenced technology adoption patterns. More experienced farmers showed higher rates of technology adoption, suggesting accumulated knowledge enhances recognition of technological benefits and willingness to implement new practices. This finding aligns with previous research indicating that experienced farmers typically possess better risk assessment capabilities and deeper understanding of farm management practices (Korir et al. 2023). Their extended exposure to various farming challenges and solutions appears to facilitate more informed decision-making regarding technology investments. Additionally, experienced farmers often have established networks and better access to information sources, further supporting technology adoption (Nyambo and Clemen 2023).

The Technology-intensive group demonstrated higher average farm size and milk production levels compared to the other group. This relationship likely reflects the interplay between land availability, resource capacity, and technology adoption. Farmers with larger land holdings typically possess greater financial resources and herd sizes, enabling investment in multiple technologies. Research indicates that this technological intensification often creates positive feedback loops, where increased production efficiency justifies further technological investment (De-Pablos-Heredero et al. 2020). Moreover, larger operations can better absorb the fixed costs associated with technology adoption through economies of scale (Nyambo and Clemen 2023). These findings align with previous studies demonstrating positive correlations between farm size, technological intensity, and productivity in dairy production systems (Korir et al. 2023).

The cultural context of technology adoption among indigenous farmers represents an important dimension of our findings. The Pastos people have maintained traditional agricultural practices for generations, creating a complex relationship with modern farming technologies. Our results suggest that technology adoption decisions within these communities are influenced by a blend of cultural values and pragmatic considerations. While specific cultural factors were not directly measured in our study, the observed patterns indicate that indigenous farmers selectively adopt technologies that align with their existing knowledge systems and cultural practices (Godoy et al. 1998). For instance, high adoption rates of pasture management techniques may reflect their compatibility with traditional Pastos land stewardship values, while lower adoption of mechanical milking might indicate preferences for maintaining traditional cattle handling practices. Further research using qualitative methods would be valuable to better understand these cultural dimensions of technology adoption.

The high rate of cooperative membership (73%) found in our study may be beneficial for dairy farming in Cumbal since agricultural cooperatives play a vital role in supporting smallholder farmers by improving their market access, increasing their bargaining power, providing technical assistance and inputs at better prices, and ultimately helping to reduce poverty through enhanced income opportunities (Jitmun et al. 2020; Neves et al. 2021; Molla et al. 2024). However, Martinelli et al. (2022) suggest that government entities could further promote producer participation in cooperatives through targeted incentives and training programs that consider cultural inclusivity and motivation.

The high proportion of dairy farmers (75%) who have received formal training in livestock production represents a significant advantage for this indigenous farming community. Training in dairy management practices has been consistently linked to improved business performance metrics, operational efficiency, and overall farm profitability (Martins et al. 2019). Research demonstrates that trained farmers typically demonstrate better herd health management, enhanced milk quality control, and more effective resource utilization (Nyokabi et al. 2021). Additionally, trained farmers are often better positioned to adopt and implement new technologies effectively, creating potential multiplier effects for farm productivity (Korir et al. 2023).

Our analysis reveals high adoption rates of artificial insemination (AI) among the surveyed indigenous farms, with 65.5% utilizing this reproductive technology. AI represents the most efficient and reliable method for genetic improvement and enhanced productivity in dairy cattle (Mwanga et al. 2019; Gowane et al. 2019; Galina and Geffroy 2023). This substantial adoption rate indicates significant technological integration within these indigenous farming systems and demonstrates farmers’ recognition of genetic improvement benefits. Nevertheless, 34.5% of farms (*n* = 187) have not implemented AI, potentially due to service accessibility limitations, economic constraints, or cultural preferences for traditional breeding practices.

Analysis of grazing management practices reveals high adoption rates (> 80%) for both portable electric fencing and fertilization applications. Electric fencing enables optimized pasture utilization through controlled grazing patterns while reducing soil compaction, overgrazing, and improving manure distribution (Morgan 2016). Similarly, chemical and organic fertilization practices have demonstrated positive impacts on pasture yields, milk production, and farm profitability (Nnahiwe et al. 2023; Souza et al. 2023). However, given the dairy farms’ location within the ecologically sensitive páramo Cumbal-Chiles complex, promoting sustainable fertilization practices remains critical for ecosystem preservation (Zapata et al. 2021; Durana et al. 2023).

The environmental dimension of technology adoption in this region warrants particular attention. The páramo ecosystem, characterized by high biodiversity and crucial hydrological functions, faces significant pressure from agricultural activities including dairy farming (Zapata et al. 2021). Technology adoption in this context presents both challenges and opportunities for ecological sustainability. While certain technologies, such as improved pasture management and electric fencing, can reduce environmental impacts by optimizing land use and preventing overgrazing, others like chemical fertilization may pose risks to water quality and ecosystem health if not properly managed (Durana et al. 2023). Future dairy development initiatives in the region should emphasize environmentally compatible technologies and practices that balance productivity goals with ecosystem conservation. Implementing agroecological approaches that build on indigenous knowledge while incorporating appropriate modern technologies could provide a pathway for sustainable intensification in this sensitive environment (Altieri and Toledo 2011).

Analysis of supplementation practices revealed widespread adoption (93%) of protein supplements and feed additives among surveyed farmers. This high adoption rate likely stems from the established correlation between supplementation and increased milk productivity, which directly influences farm income (Auldist et al. 2016). Farmers implement targeted supplementation strategies based on key production parameters including breeding status, milk yield, lactation stage, and body condition scores (Hills et al. 2015).

The dairy sector in Cumbal shows significant heterogeneity, with Technology-intensive adopters demonstrating higher modernization levels and better economic outcomes, while Technology-traditional adopters maintain more traditional practices. Geographic location emerged as a crucial factor in technology adoption and farm performance. These findings suggest the need for differentiated support programs considering both spatial and cultural dimensions when promoting dairy sector development in indigenous territories.

Although our study did not include detailed cost-benefit analyses of adopted technologies, the observed correlation between technology adoption and increased milk production suggests potential economic benefits. Future research should systematically evaluate the economic viability and return on investment for various technologies in indigenous contexts, considering both direct costs (implementation, maintenance) and indirect benefits (reduced labor, improved product quality, market access). This economic dimension is critical for developing appropriate technology dissemination strategies and supporting informed decision-making by indigenous farmers considering technological investments (Läpple and Thorne 2019; Nnahiwe et al. 2023).

Additionally, future research using mixed methods would provide valuable insights for understanding the cultural factors influencing technology adoption decisions, including traditional knowledge integration, community decision-making processes, and indigenous perspectives on agricultural modernization. Longitudinal studies examining how these production systems evolve over time would provide valuable insights for developing culturally appropriate interventions that can enhance productivity while preserving indigenous farming traditions.

## Data Availability

The data presented in the current study are available on request from the corresponding author.
